# Channel-independent recreation of artefactual signals in chronically recorded local field potentials using machine learning

**DOI:** 10.1186/s40708-021-00149-x

**Published:** 2022-01-07

**Authors:** Marcos Fabietti, Mufti Mahmud, Ahmad Lotfi

**Affiliations:** 1 Department of Computer Science, Nottingham Trent University, Clifton Lane, NG11 8NS Nottingham, UK; 2 Medical Technologies Innovation Facility, Nottingham Trent University, Clifton Lane, NG11 8NS Nottingham, UK; 3 Computing and Informatics Research Centre, Nottingham Trent University, Clifton Lane, NG11 8NS Nottingham, UK

**Keywords:** Local field potential, Artefacts, Neural networks, Machine learning, Neuronal signals

## Abstract

Acquisition of neuronal signals involves a wide range of devices with specific electrical properties. Combined with other physiological sources within the body, the signals sensed by the devices are often distorted. Sometimes these distortions are visually identifiable, other times, they overlay with the signal characteristics making them very difficult to detect. To remove these distortions, the recordings are visually inspected and manually processed. However, this manual annotation process is time-consuming and automatic computational methods are needed to identify and remove these artefacts. Most of the existing artefact removal approaches rely on additional information from other recorded channels and fail when global artefacts are present or the affected channels constitute the majority of the recording system. Addressing this issue, this paper reports a novel channel-independent machine learning model to accurately identify and replace the artefactual segments present in the signals. Discarding these artifactual segments by the existing approaches causes discontinuities in the reproduced signals which may introduce errors in subsequent analyses. To avoid this, the proposed method predicts multiple values of the artefactual region using long–short term memory network to recreate the temporal and spectral properties of the recorded signal. The method has been tested on two open-access data sets and incorporated into the open-access SANTIA (SigMate Advanced: a Novel Tool for Identification of Artefacts in Neuronal Signals) toolbox for community use.

## Introduction

When recording neural signals, other electrical sources either instrumental or physiological may distort the process. They are commonly known as artefacts, and their identification and removal are of importance to further analyse and infer insights from them. They produce longer review times [5], misdiagnosis of diseases or brain conditions (as in the diagnosis of Schizophrenia, sleep disorders and Alzheimer’s disease [32]) or produce false alarms (as in generating false alarms for brain seizures [49]). One of the most common approaches is to discard the affected epochs; however, it causes information loss and sharp discontinuities in the signal. This can impact the use of a brain–computer interfaces as the system cannot obtain the decoding results during the corresponding time. Another case would be where the signal is not meant to be evaluated by a physician but instead processed by an algorithm, causing distortions in the output.

As an alternative to keeping or discarding the corrupted segments, there are techniques that allow for their removal, such as filtering, template subtraction, or advanced computational techniques. Invasively recorded signals are less susceptible to external artefacts, but must be processed nonetheless. Local Field Potentials (LFP) are low-pass filtered signals of the extracellular electrical potential recorded in deeper layers of the brain through micro-electrodes [28, 34]. In case of LFP, several signal analysis and processing toolboxes offer a range of computational techniques for artefact removal including signal filtering for unwanted components, removal of power line noise, rejection of channel with incompatible interference, automated removal of noisy signal components etc. [18, 26, 27, 38]. Most of these techniques involve the removal of segments that have been corrupted by the noise/artefacts and this often distorts the overall integrity of the signal, which is undesirable in cases, where further processing relies on the completeness of the signal.

To recover the original signal with the aim of preserving the information, machine learning (ML) techniques have been applied to this task. These techniques gather information presented to them to construct a model which can be used to make inferences about unseen data, and have been widely used in diverse fields, for example: outlier detection [11, 14, 57], data mining of biological data [29, 30], detection of diseases [35, 39, 47, 50, 59], elderly monitoring[2, 22, 37], financial forecasting [40], image processing [3, 45],natural language processing [44, 56] and monitoring patients [1, 52]. Among the many ML methods, deep neural networks stand out. Their design was inspired by the biological counterpart, and they allow for non-linear processing of information.

Within the ML-based solutions, the research found in the literature commonly employs multi-channel solutions. This generates a shortcoming, as they are invalidated if the number of affected channels are more than the ones not affected, or if a global artefact appears. Therefore, channel independent solutions are needed, which can be used in low-channel applications and expanded as needed. This work extends the conference contribution presented at the 14th International Conference on Brain Informatics [12]. In that work, a deep learning-based approach was proposed as an artefact removal module for the SANTIA (SigMate Advanced: a Novel Tool for Identification of Artefacts in Neuronal Signals) open-source artefact removal toolbox [13]. SANTIA allows the detection and subsequent removal of artefacts in LFPs by replacing the artefactual segments with signals generated using a single-layer Long–Short-Term Memory (LSTM) network. This current work extends the conference work by validating and testing it using a second data set. It also reports the robustness of the method by expanding the methodology to a more complex network architecture as well as a non-ML method for comparison. Overall, this extended version provides an in-depth description of the methodology and describes the implementation of the improvements.

The remainder of this paper is organised as follows: Section 2 describes the state-of-the-art for artefact detection and removal, Section 3 presents the methods proposed in the current work, Section 4 shows the usage of the proposed artefact removal methods after their incorporation into the SANTIA toolbox, Section 5 reports the results obtained on publicly available open-access data sets, and finally, Section 6 provides the conclusion of the work.

## Related work

**Fig. 1:**
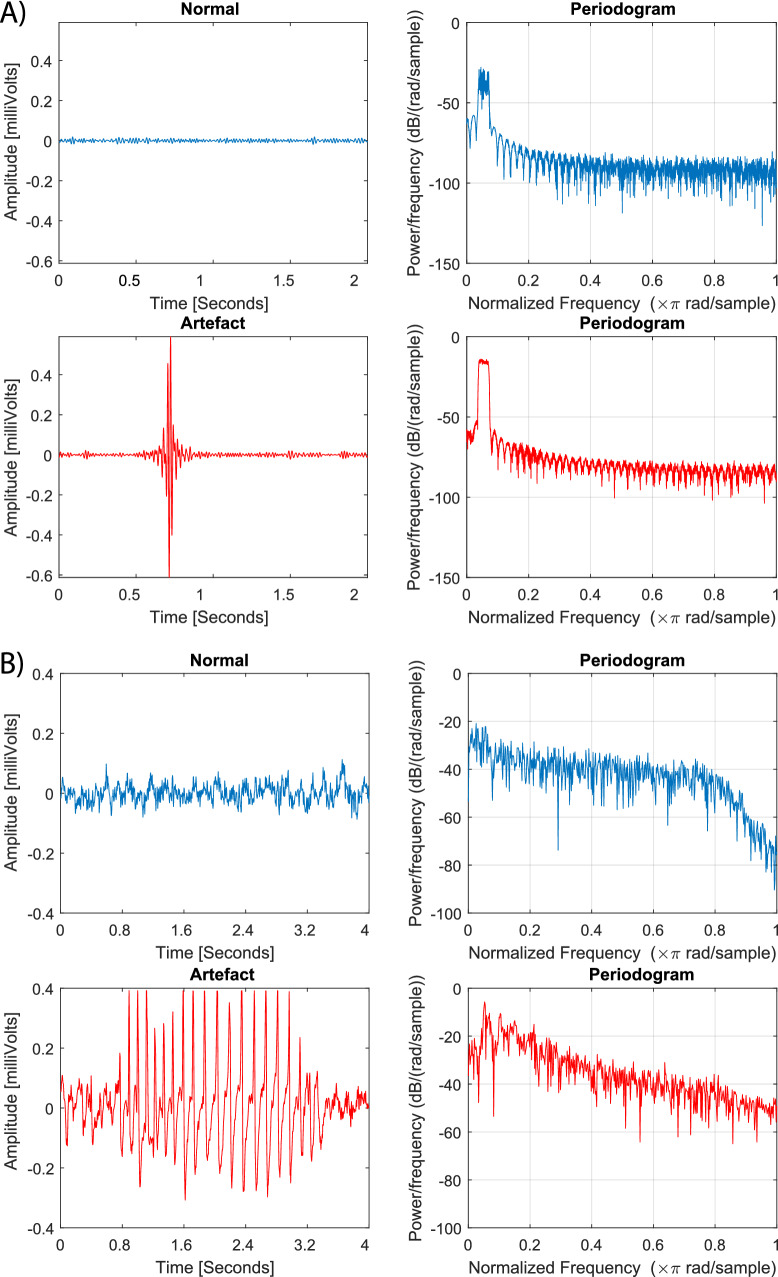
Examples of signal segments with (red) and without (blue) artefacts along with their respective periodograms for data set 1 (**a**) and data set 2 (**b**)

When attempting to remove artefacts, there are several computational approaches that are typically used. For illustration’s sake, Fig. 1 displays signal segments with and without artefacts, alongside their frequency components of the two data sets used in this paper (1a represents the data set in section 3.2 and 1b shows a representative artefact from the data set described in section 3.3). Brief discussions about these existing approaches are provided in the following paragraphs.

*Regression* A regression method begins by defining the amplitude relationship between a reference channel and a neural signal using transmission factors, then removing the estimated artefacts from the signal [55]. In a single-channel approach without a reference channel, this approach is not possible.

*Adaptive Filtering* To apply adaptive filtering, a reference channel is given as one of the inputs to the filter, so the degree of artefactual contamination in the neural signal is measured by iteratively changing the weights according to the optimisation method and then removed [23]. As with regression, the lack of a reference channel invalidates applying this approach.

*Template subtraction* When artefacts have a unique shape, as they come from a specific source, it can be approximated and subtracted to restore the neural signal [36]. As a result of the variance of the shapes of the artefacts in the data sets, as they can be of different unidentified sources, make it impossible to accurately subtract it without introducing further error.

*Inter-channel interpolation* When a channel in an array is impacted locally by an artefact, that segment can be replaced using the average or other methods that take into consideration the surrounding channels, which isn’t possible in a single channel approach [4].

*Decomposition* One major drawback of decomposition methods (e.g., wavelet, empirical mode) is that they cannot remove artefacts completely if the spectral properties of the measured signal overlap with the spectral properties of the artefacts [20]. In the data sets, artefactual segments manifest in the same bands as the physiological signal.

*Blind source separation* Blind source separation is a popular method for removing artefacts in neuronal signals and includes methods, such as independent component analysis, canonical correlation analysis and principal component analysis [21]. However, these methods assume that the number of artefact sources should at least be equal to the number of channels, limiting the single channel applications.

This is clear from the above discussion that most traditional methods fail to recreate the artefactual region of the signal. To this end, we propose an alternative to discarding the segment, which is to replace it with a model-generated sequence of “normal” behaviour of the signal. This way, subsequent analyses of the signal are not hampered by the absence of segments. To demonstrate the accuracy of the model-generated replacement segments, we applied it to two completely different publicly available data sets (see sections 3.2 and 3.3). From a perspective of restoration of missing values in neuronal signals, there have been cases of both ML or non-ML approaches in electroencephalogram (EEG) signals.

From the first group, Svantessona et al. [53] trained a convolutional neural network (CNN) with 4, 14 and 21 EEG channel inputs to up-sample to 17, 7 and 21 channels, respectively. A visual evaluation by board-certified clinical neurophysiologists was conducted, and the generated data was not distinguishable from real data. On a similar approach, Saba-Sadiya et al. [46] employed a convolutional autoencoder, which takes as an input a padded EEG electrode map during 16ms (8x8x8 tensor) with 1 occluded channel, which is expected as the output. They compared it to spherical splines, euclidean distance and geodesic length methods, outperforming them and showing the method is able to restore the missing channel with high fidelity to the original signal. Finally, Thi et al. [54] utilised a linear dynamical system (Kalman Filter) to model multiple EEG signals to reconstruct the missing values. This method showed 49% and 67% improvements over singular value decomposition and interpolation approaches, respectively.

In the second group, there are published papers, such as de Cheveigne and Arzounian [8] and Chang et al. [6]. In [8] authors have detected EEG and magnetoencephalography artefacts by their low correlation to other channels, and replaces them with the weighted sum of normal channels, a method called ’Inpainting’. On the other hand, Chang et al. employed artefact subspace reconstruction on twenty EEG recordings taken during simulated driving experiments, in which large-variance components were rejected and channel data were reconstructed from remaining components improving the quality of a subsequent independent component analysis decomposition. Sole-Casals et al. [51] evaluated the performance of four tensor completion algorithms and average interpolation across trials on missing brain–computer interface data (across 6 channels and segments), and evaluated the reconstruction by the performance of machine-learning-based motor imagery classifiers.

Overall these approaches rely on the information from other channels of the arrays, which fails when a global artefact is present, the number of affected channels are more than the ones not affected, or they have poor quality. For those situations, we propose the usage of the surrounding information of the affected channel instead to accurately replace the segments affected by artefacts via the use of deep learning.

## Methods

In this section, the ML methods as well as the data sets used are described.

### Machine learning model

**Fig. 2:**
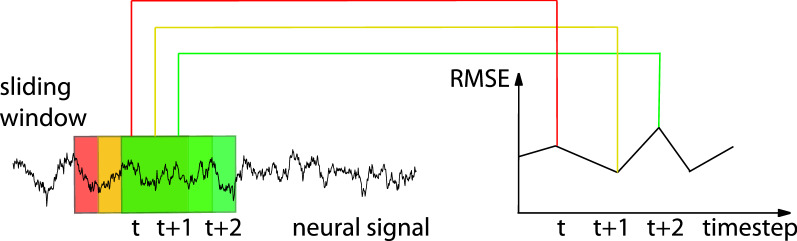
Sliding window approach diagram, based on [7]

We hypothesise that by training an LSTM network to reliably forecast artefact-free data, it may be successfully utilised to substitute artefactual sections of signals when information from other channels has been corrupted and cannot be used to approximate its real behaviour. Figure 2 shows how an LSTM network was trained to predict typical behaviour using a sliding window method. The sliding window approach consists of employing data at a time *t* to predict the value at $t+1$, and then uses the new predicted value when forecasting the value at $t+2$.

The neural network architecture was chosen due to the known capabilities of Recurrent Neural Network (RNN), specifically LSTM, in recognising patterns from sequential data. Kim et al. [25] has proven that it is possible to predict the behaviour of local field potentials from 10 to 100 ms forward in time via the use of a regressive LSTM network. A similar approach was established by Paul [42], who used a stacked LSTM to forecast a single point of an EEG signal by feeding the previous 70 ms. Their test data was composed of 9 subjects, in which they achieved correlation coefficients of over 0.8 across all of them. In addition, there have been recently reported applications of LSTM in artefact detection [15, 19, 24, 31] as well as RNN in artefact removal [10, 41, 43, 48]. The LSTM cells include a forget gate which decides what information is kept and what information is discarded from the cell state. If the value of the forget gate $f_t$ or *f*(*t*) is 1, the relevant information is saved, but if the value of the forget gate is 0, it is forgotten. Equation 1 shows the mathematical expression of this specific LSTM cell.

1 $$\begin{aligned} {\begin{matrix} f_{t}=\sigma (W_{fh}h_{t-1}+W_{fx}x_{t}+b_{f}),\\ i_{t}=\sigma (W_{ih}h_{t-1}+W_{ix}x_{t}+b_{i}),\\ \tilde{c}_{t}= tanh (W_{\tilde{c}h}h_{t-1}+W_{\tilde{c}x}x_{t}+b_{\tilde{c}}),\\ c_{t}=f_{t}\cdot c_{t-1}+i_{t}\cdot \tilde{c}_{t},\\ o_{t}=\sigma (W_{oh}h_{t-1}+W_{ox}x_{t}+b_{o}),\\ h_{t}=o_{t}\cdot tanh(c_{t}) \end{matrix}} \end{aligned}$$

where the variable $x_{t}$ is the input vector, *W* holds the weights, *b* is the bias and $\sigma$ is the sigmoid function. In addition, $f_{t}$ is the forget gate, $i_{t}$ is the update gate, $\tilde{c}_{t}$ is the cell input, $c_{t}$ is the cell state, $o_{t}$ is the output gate and $h_{t}$ the hidden state or output vector of the cell at time *t*.

The testing set was used to calculate the root mean squared error (RMSE), as defined in Eq. 2, of the output over an unseen segment.

2 $$\begin{aligned} RMSE=\sqrt{\frac{\sum _{i=1}^{S}\sum _{j=1}^{N}(x_{ij}-\hat{x}_{ij})^{2}}{N}} \end{aligned}$$

where ${x}_{ij}$ is a forecasted data point, $\hat{x}_{ij}$ the real value of the LFP at that data point, *S* is the output sequence length and *N* the number of examples in the test set. This was chosen over the mean absolute percentage error (MAPE) due to the fact that the signal has been zero centred during the pre-processing, so the number of zero crossings a segment has is significant, which distorts the MAPE as it takes an undefined value in those points and they must be removed. Matlab’s Deep Learning Toolbox [33] was used to build and train the network of LSTM cells. The LSTM models were made up of the following layers: an input layer, a hidden layer equal to one-tenth of the input, and an output layer equal to the number of predicted points. For comparison, we trained a more complex architecture composed of convolutional and recurrent layers CNN-LSTM described in Table 1. The optimisation algorithm used was Adam, with an initial learning rate of 0.0001, momentum of 0.9 and a batch size of 516 for the first data set and 128 for the second data set, due to having a smaller sample size. The loss function of the regression layer was the half-mean-squared-error of the predicted responses for each time step, not normalised by N:

3 $$\begin{aligned} loss=\frac{1}{2S}\sum _{i=1}^{S}\sum _{j=1}^{N}(x_{ij}-\hat{x}_{ij})^{2} \end{aligned}$$

where ${x}_{i}$ is a forecasted data point, $\hat{x}_{i}$ the real value of the LFP at that data point, *S* is the output sequence length and *N* the number of examples in the training or validation set.

**Table 1:** CNN-LSTM structure

Layer	Type	Description
1	sequenceInput	–
2	sequenceFolding	–
3	convolution2d	size=5, filters=32, dilation=1
4	batchNormalization+elu	–
5	convolution2d + elu	size=5, filters=32,dilation=2
6	convolution2d + elu	size=5, filters=32,dilation=4
7	convolution2d + elu	size=5, filters=32,dilation=8
8	convolution2d + elu	size=5, filters=32,dilation=16
9	averagePooling2d	size=1,stride=5
10	sequenceUnfolding	with flattening
11	gru	128
12	lstm	64
13	dropout	0.25
14	lstm	32
15	dropout	0.25
16	regression	–

To have a performance reference, the linear approximator autoregressive moving average with extra input (ARMAX) was applied on the same testing and model evaluation data. Following the description by Yan et al. [58], given a LFP time series ${(X_{t}, y_{t})}$ for $t = 1$ to *N*, where $X_{t} = t(x_{t1},x_{t2},..., x_{tk})$ is the input vector at time *t* with *k* elements and $y_{t}$ is the corresponding neuronal activity voltage at time *t*, this model approximates a polynomial equation, written as:

4 $$\begin{aligned} A(q)y_{t}=\sum _{i=1}^{k}B_{i}(q)x_{ti}+C(q)e(t) \end{aligned}$$

where *A*(*q*), *B*(*q*) and *C*(*q*) are the polynomials expressed with a time shift term $q^{-1}$ shown in Eq. 5 and *e*(*t*) is the white-noise disturbance value.

5 $$\begin{aligned} {\left\{ \begin{array}{ll} A(q)=1+a_{1}q^{1}+...+a_{n_{a}}q^{n_{a}}\\ B_{i}(q)=1+b_{1i}q^{1}+...+b_{n_{bi}}q^{n_{bi}+1}\\ C(q)=1+c_{1}q^{1}+...+c_{n_{c}}q^{n_{c}} \end{array}\right. } \end{aligned}$$

Here, the hyper-parameters $n_{a}, n_{b}, n_{c}$ denote the orders of the ARMAX model’s auto-regressive part, external input vector with k elements and moving average, respectively. Finally, $a_{i}, b_{ik}$ and $c_{i}$ are the polynomial coefficients determined using polynomial curve fitting. Having described the methodology, we proceed to describe the data sets used to evaluate it.

### Data set 1

Open-access data was utilised to evaluate the toolbox [16]. The data set is linked to an article that provides a detailed report on the recordings and trials [17]. Male Long Evans rats (280 to 300 g) trained to walk on a circular treadmill were used to generate the recordings. The obtained LFPs were sampled at a rate of 2 kHz, and then pre-processed first by low-pass filtering them, second by amplifying times a thousand and finally applying a bandpass filter from 0.7 to 150 Hz.

**Table 2:** Guide to determine the best channels and epochs to use of baseline walk and rest recordings in medial prefrontal cortex (mPFC) and the mediodorsal (MD) thalamus, as mentioned in the file named “Coherence Phase Plot Guide”. Column 1 denotes animal id, columns 2 and 3 shows two good channels of the mPFC recordings and coumns 4 and 5 of the MD recordings. Finally, columns 6 and 7 show the range of artefact free epochs during walking and at resting, respectively

Rat	mPFC chan1	mPFC chan2	MD chan1	MD chan2	Walk epoch	Rest epoch
KF9	5	6	3	7	960–1160	3780–3820
KF10	3	4	3	8	670–860	1260–1390
KF14	2	6	5	7	740–940	3350–3550
KF15	3	4	5	7	450–640	1600–1700

To evaluate the toolbox, a subset of the repository composed of baseline recordings (i.e., before Ketamine administration) was used. The baseline recordings included at least two 5-min counter-clockwise walking loops on a slow-moving treadmill and two 40-s rest intervals free of artefacts. Artefact-free intervals of 100 s in treadmill-on epochs and 40–100 s periods in treadmill-off epochs were classified using visual inspection and recorded motor activity, which are detailed in Table 2. The threshold power value for each channel was calculated using these labelled artefact-free epochs, defined as the maximum power of windows of 50 ms duration within them, where the window length was chosen based on prior classification findings.

One-second artefact-free windows were extracted for each of the rodents and then aggregated to a cross-subject data set, which was divided into training (80%), validation (10%) and testing (10%) sets. Out of the training and validation sets, 54 data sets were constructed based on the length of the input from 0.1 to 0.9 in 0.1 increments and the prediction of posterior 1, 5, 10, 25, 50, and 100 data points. To be able to compare the different forecasting output sizes, the test set was used to evaluate the performance over 0.1 s (i.e., 200 points at 2 kHz) of unseen data.

### Data set 2

**Table 3:** Total time of awake segment per rodent of the data set

rodent	recording ID	longest awake segment (seconds)	Totals (seconds)
AsiagoBleu	180626	3508	9500
	180627	1776	
	180628	1728	
	180629	2488	
bobmarley	102819	4236	18196
	102919	4776	
	103119	4228	
	110319	3012	
	110419	1944	
cheaptrick	110619	2480	17240
	110719	11608	
	111319	3152	
EZBrie	180707	4320	19064
	180708	4320	
	180709	3228	
	180710	7196	
FetaMozz	180628	7124	17340
	180629	6860	
	180630	3356	
Manchego	180620	4456	16156
	180621	5396	
	180622	2188	
	180623	4116	
MuensterMonty	180720	3408	26956
	180721	7648	
	180723	5024	
	180726	3380	
	180727	7496	
NachoGouda	180705	2656	9140
	180706	2496	
	180707	2136	
	180708	1852	
neilyoung	111719	2840	26516
	111819	3320	
	111919	3272	
	112119	3284	
	112219	4148	
	112619	4040	
	112819	3200	
	112919	2412	

A second open-source data set [9] was used to test the methodology. We have selected this data set based on the amplitude of the artefacts, which were ranging between 0.15% and 13.48% of the recordings, as highlighted by the authors on the related work. The open-access data set is composed of uninterrupted baseline recording days for sleep research, where local field potentials were recorded from 9 male Sprague–Dawley rats (3–4 months). The data set contains LFP that were acquired at the prefrontal and cortex parietal cortex, sampled at 250 Hz. Recordings were cut into 4-s long epochs and labelled depending on the state of the animal (awake, rapid eye movement, or non-rapid eye movement sleep).

It is worth noting that the data set has intra-subject variability, as these recordings range from 3 to 8 consecutive days (out of 40 that are not shared), as well as inter-subject variability, since it has twice the number of subjects as the first data set. Furthermore, there are differences between states, such as high-frequency components which may distort the detection and removal of artefacts. Therefore, to reduce the variability we extracted the longest awake period of each day (see Table 3), and chose the rodent with the longest consistent awake recordings (i.e., rodent ‘MuensterMonty’). The final data set is composed of the recordings of one rodent during the awake state across five recording sessions for a total of 26956 s.

Afterwards, we measured the signal’s power with a 1-s moving window, and if it exceeded the threshold defined manually defined in the toolbox, the segment was classified as artefacts. Due to the small sampling rate, we extracted 4-s non-artefact segments from each of the recordings. These were divided into training (80%), validation (10%) and testing (10%) sets. Out of the training and validation sets, 15 data sets were constructed based on the length of the input from 1, 2, or 3 s and the prediction of posterior 1, 25, 50, 125, and 250 data points. To be able to compare the different forecasting output sizes, the test set was used to evaluate the performance over 1 s (i.e., 250 points) of unseen data.

## Implementation

The SANTIA toolbox is composed of four units that carry out different tasks on the neural recording files, these are: data labelling, neural network training, classifying new unlabelled data, and artefact removal. While the first three are used for artefact detection, the first and fourth units are used for artefact removal. The labelling unit performs the following tasks:data loading, scaling, reshaping, channel selection, labelling, saving and 2D display. On the other hand, the fourth unit is composed of: data loading, normal segments extraction, hyper-parameter setting, network selection, network train, test set visualisation, replace segments, plot replaced channels, and saving.

The toolbox is available for download directly from the Github repository^1^. The GUI allows quick access to all modules when the toolbox has been launched. We highlight that SANTIA is not a library of functions with a GUI added to make access easier but instead is a generic environment built on a single interface with individual features implemented. Interactions with the GUI are made by selecting functions, settings, and keyboard inputs, which are processed in the back-end. A check procedure runs before each function to ensure that the user hasn’t skipped a step or failed to include all of the needed inputs or parameter selections. This is done to minimise both the risk of human mistakes and the amount of time consumed. If the user has a question, tool-tips with a brief explanation display when the pointer is held over a component of the GUI.

We now proceed to describe the aforementioned units relevant to the task as well as the outputs produced.

### Data labelling

The first step is loading the neural recordings, which is done with the import wizard launched by the ‘Load Signals’ button of the first unit, as a matrix with m number of channels and n number of data points for each channel. ASCII-based text (e.g., .txt, .dat, .out, .csv), spreadsheet files (e.g., .xls, .xlsx, .xlsm) , and Matab files (e.g., .set, .mat) are the formats that are compatible with the toolbox. To structure the data, the user must provide the sampling frequency in Hz and the window duration in seconds. The options for data scaling are available to avoid the common incorrect magnitude annotations.

A function to structure the data is called via the ‘Generate Analysis Matrix’ button, which takes in the aforementioned inputs. The following step consists of labelling the data, carried out by giving segments whose power exceeds a user-defined threshold a binary label. The toolbox allows for three options, either of which the user can use to their preference. The first is table that hold the segment power in the first column and the values of the signal in the subsequent columns. The user may sort any column to define a value which divides both classes in the optimal way, and visualise any segment they select. The second option is the ‘histogram threshold’, where a histogram of the segments’ power shows the distribution, and the user can select with a slider the cutoff value, or visualise a segment.

As an alternative, the threshold values can be typed into the table displayed on the module. Once all channels have been filled, the signals are labelled and saved as a standardised struct, which includes the original filename, the structured data with its labels, the sampling frequency, window length, the scale, and the threshold values. The purpose of the format is to allow users to select and contrast the various data sets they build, due to different window lengths or threshold values they may have chosen. Users can see when each stage has been finished with the help of text in the ’Progress’ banner, which is duplicated across each unit.

### Artefact removal

The initial step of this unit is to load the structured file mentioned above. Once complete, the user must input the duration of artefact-free segments they wish to extract from the file to train the model. A progress bar indicates the progress of the extraction, followed by a notification of the number of segments extracted upon its completion. The following step is the configuration of the input and output of the model, with the option of selecting either data points or milliseconds as units. They must also input how to split the data for training, validation, and test sets, as they are crucial to avoid over-fitting.

**Fig. 3:**
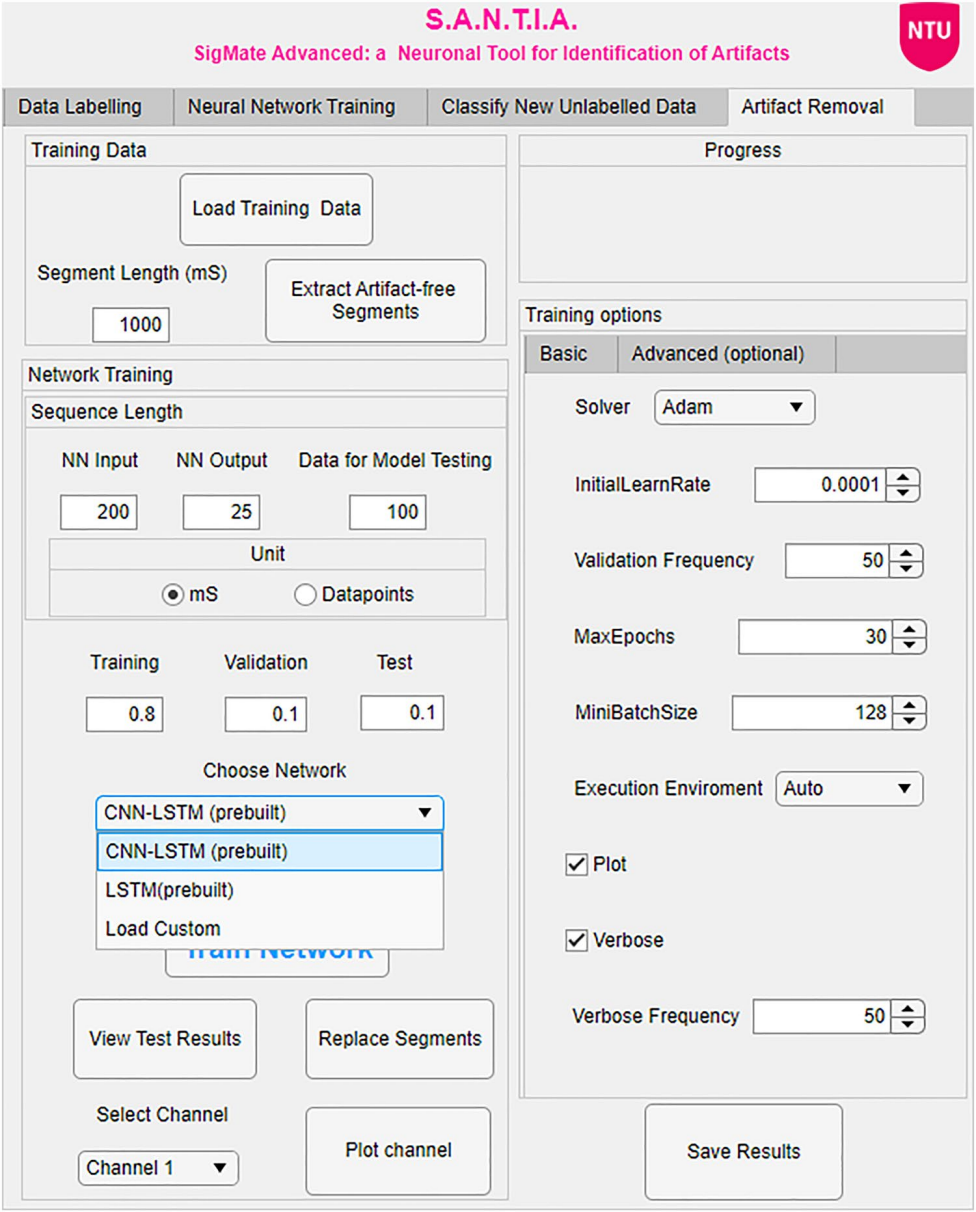
Architecture selection option of the artefact removal module in the SANTIA toolbox

For the third step, a new option has been incorporated which allows users to make use of the CNN-LSTM architecture presented in this work, the previously reported LSTM or for the user to load his/her custom set of layers, as shown in Fig. 3. The file must contain a Layer-type variable, in other words, layers that define the architecture of neural networks for deep learning without the pre-trained weights. These can be modified via console or the Deep Network Designer Toolbox, for more information, we direct the reader to the Mathworks page^2^.

A side panel allows the customisation of training hyper-parameters, such as the validation frequency, max epochs, verbose, mini-batch size, and others. These intentionally mirror the ones available in the Deep Network Designer, making it easier to familiarise with it. The training process is run by clicking on the ‘Train Network’ button, which loads all the user-defined inputs so far and generates a training plot for the user to evaluate the process and do an early stopping if required.

A pop-up notification alerts the user of the root mean square error of the test set, and the user can visualise the examples of the test set in contrast to their forecast. The user can either adjust the network and training parameters to get a desirable result, and once obtained, they can proceed to the last step. This consists of swapping the windows labelled as artefacts for the network’s forecast, where a progress bar is displayed to show the advancement. The newly obtained signals can be visualised by first selecting which channel to display and the ‘Plot Channel’ Button. The last step is to save all the obtained information in the form of a struct with data’s filename, the trained network, the training information, the test set’s RMSE, the test set original, and replaced segments and the data with the artefactual data removed, where the user sets the file name and directory to store it.

### Performance evaluation

**Fig. 4:**
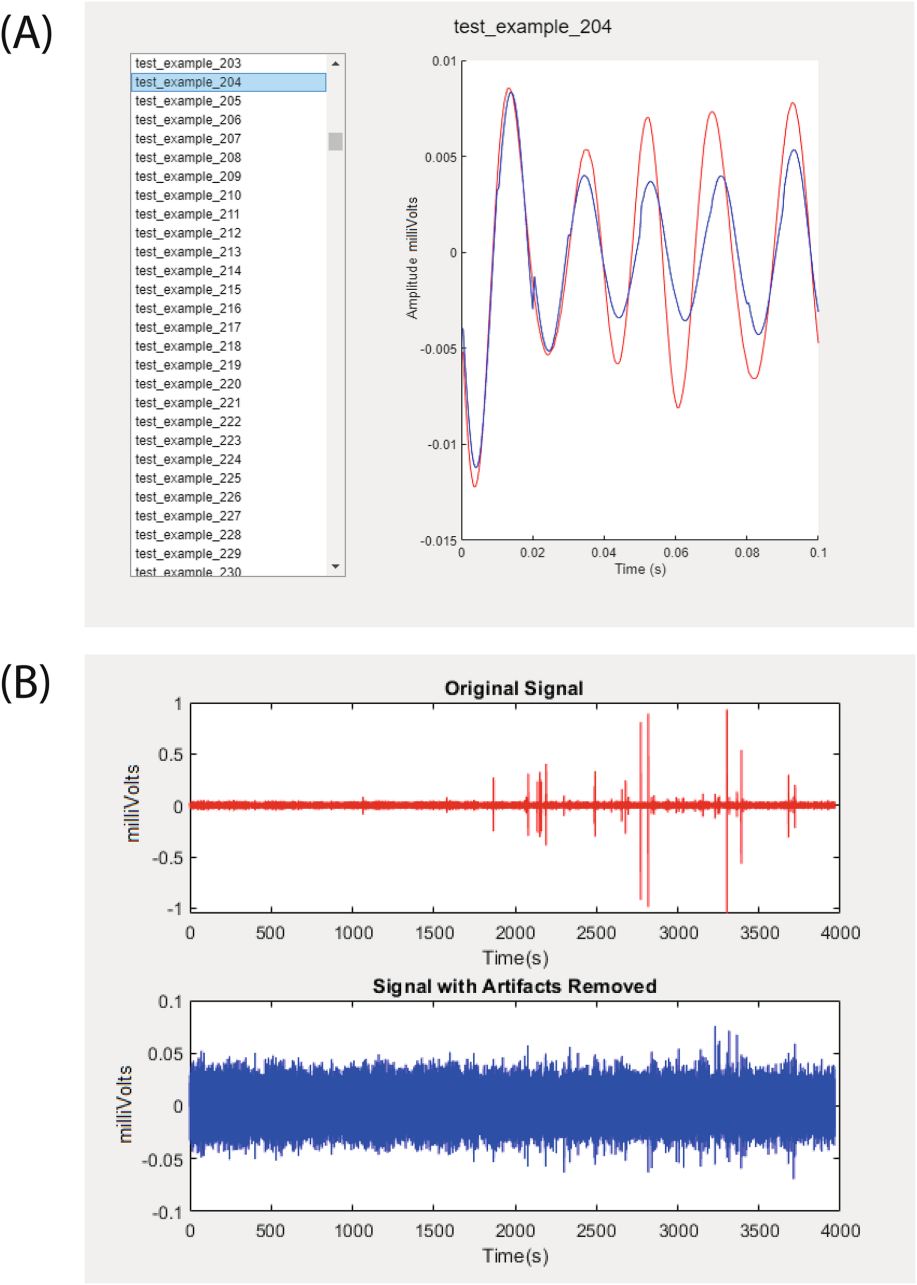
Visualisation of the test set (**a**) and comparison of the original signal with artefacts removed (**b**) are the outputs of the artefact removal module. The original signal appears in red in both outputs, while the predicted or artefact-free signal appears in blue

In order for the user to compare the different models, and adapt the network size, type or hyperparameters, the toolbox creates several windows. These are showcased in Fig. 4, which displays examples of the outputs of ‘View Test Results’ (A) and ‘Plot Channel’ (B). In the upper sub-figure, we showcase an element of the test set in red in contrast to the forecast of the CNN-LSTM network in blue. In this particular example, while the forecast of the first peak is nearly identical to the signal the following peaks have slightly less amplitude, which can be attributed to the fact that they are taking in the previous forecasts of the network. The sub-figure below showcases a channel before (red) and after removal (blue). The high amplitude artefacts which spanned 2 mV peak-to-peak have been removed and replaced by 50 ms windows, and now the channel shows a uniform range of $\pm 0.05$ mV, indicating the success of the methodology.

## Results

### Data set 1

**Fig. 5:**
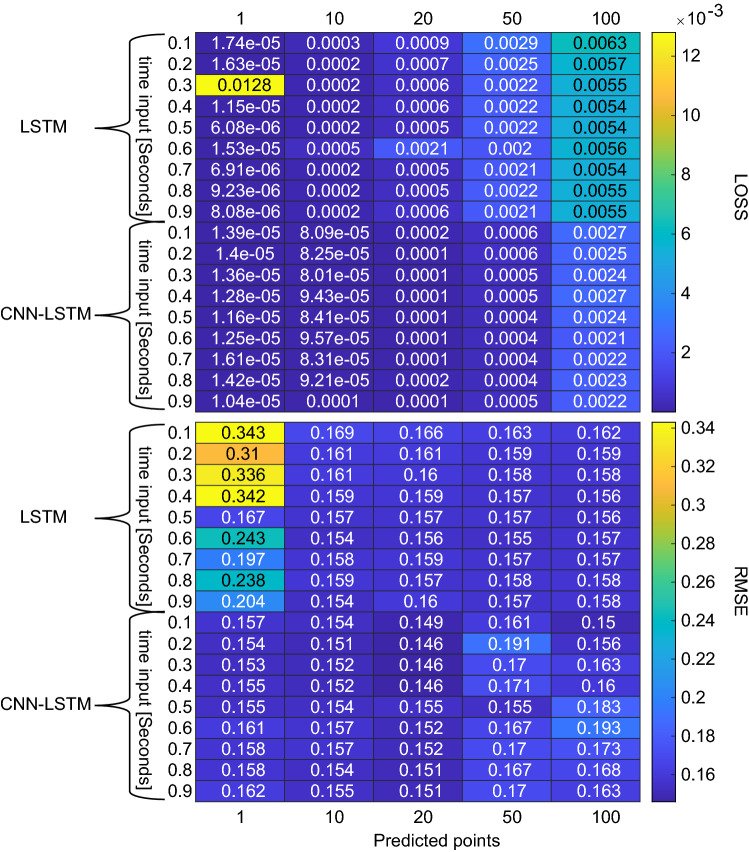
Validation loss (top) and test set RMSE (bottom) of each predicted points (column) vs time input (row) of the LSTM and CNN-LSTM models trained with data set 1

Figure 5 shows performance of the 54 LSTM models in the form of validation loss and test set RMSE over 100 ms. In regards to the output of the network, the test performance improves from single value predictions to the fifty points one and then remains constant. In regards to the time input, larger sequences above 0.6 s don’t present any major performance improvements. The best performing LSTM model is the 600 ms input and 10 points prediction model with an RMSE of 0.1538.

On the other hand, out of the 54 CNN-LSTM models, the best performance is achieved with an output of 20 data points across all inputs, while the worst performances are achieved with 50 or 100 output points. Overall, the performance of the CNN-LSTM is better than the LSTM models, with the best score being 0.1463 of the 200 ms input and 20 points prediction model.

To confidently prove the effectiveness of this method, it has been compared to ARMAX. The ARMAX was given the same 200 ms examples for defining the model and the 100 ms to calculate the RMSE, which achieves a performance of 0.1449. This indicates a slightly better performance than the neural networks; however, we must factor in that the signals have been significantly low-passed filtered and the signals have a near-sinusoidal shape. If used on a different set that retains higher frequency components, the performance of the ARMAX model would be challenged, as we will show on the next data set. Besides the forecasting ability of the models, we evaluated as well the computational time by forecasting 0.1 s of recording and averaged over 100 iterations.

**Table 4:** Performance comparison for forecasting methods

Method	RMSE	Time (s)
LSTM	0.1538	0.0433
CNN-LSTM	0.1456	0.0547
ARMAX	0.1449	1.5425

**Fig. 6:**
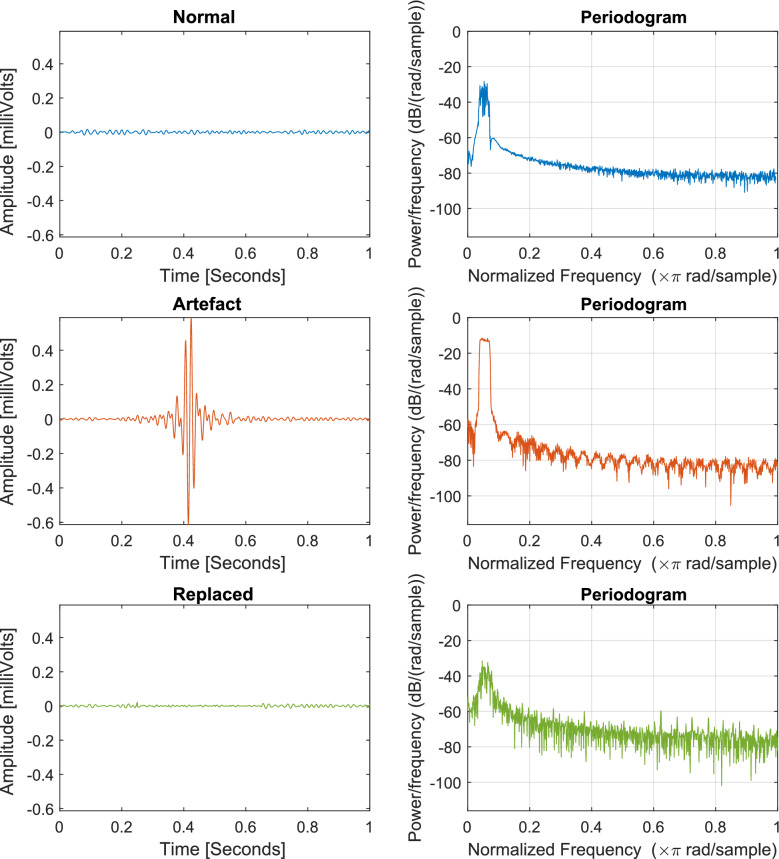
Examples of normal (blue), artefactual (red) and replaced-segments signals (green) alongside their periodograms for data set 1. The method has been able to recreate the normal signal, both in amplitude as in spectral properties

Results are depicted in Table 4, where the neural network method outperforms ARMAX significantly in computational time. The time difference is mainly due to the fact that the ARMAX needs to estimate the grades of the polynomials every new sequence for accuracy, unlike the CNN-LSTM that is able to forecast very rapidly, once it has been trained. All models were tested on a general-purpose Alienware m17 r4 laptop consisting of 32 gigabytes of RAM and Intel®$\hbox {Core}^{\mathrm{TM}}$ i9-10980HK CPU @ 2.40 GHz processor. With both metrics, i.e., RMSE and computational time, we choose the CNN-LSTM as the best compromise between the two. Having defined the best model, a total of 7275 1-s artefactual segments were extracted from the data of the rodents, with the condition that the first 200 ms had to be artefact-free. The forecast produced by the network replaced every 50 ms window labelled ‘artefact’ in each segment, which in turn was used as part of the input if the following window also shared the same label.

**Fig. 7:**
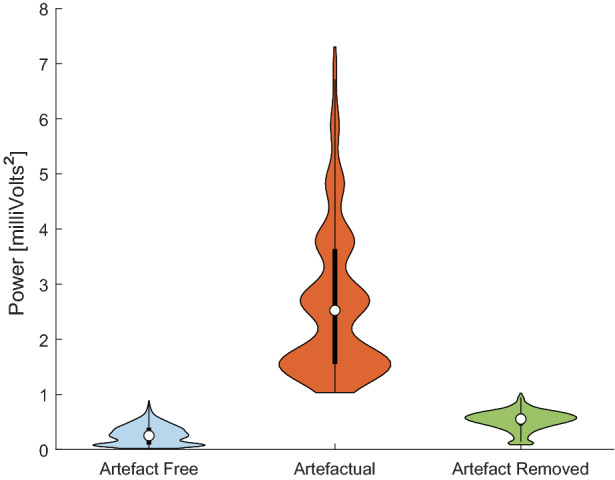
Violin Plot of power in the normal (blue) 1 s segments, artefactual segments before (red) and after (green) processing from data set 1. The method has reduced the power of the artefactual segments to similar values to the artefact-free segments

The first comparison of the results is done through visual inspection. Examples of normal, artefactual, and replaced-segments signals alongside their periodogram are illustrated in Fig. 6. The new signal after the processing had had its high amplitude artefact removed, demonstrating the method’s success. This can also be observed in the periodogram, where the artefactual example possesses a low-frequency component that exceeds the $-20$ dB, but the physiological as well as the processed signal have a power of approximately $-40$ dB.

In regards to segment’s power, Fig. 7 shows the violin plot^3^ distribution of the three groups: the normal segments, artefactual segments and after replacing them. The method has been successful in replacing the high power artefactual segments with ones that resemble normal activity. While the median is higher than the artefact-free, the distribution has shifted considerably to lower power levels. The presence of high-power segments indicates a shortcoming of the method, where surrounding information has high power, but only one or two windows do exceed the defined threshold, so the total sum of the processed segment still has a high value.

### Data set 2

**Fig. 8:**
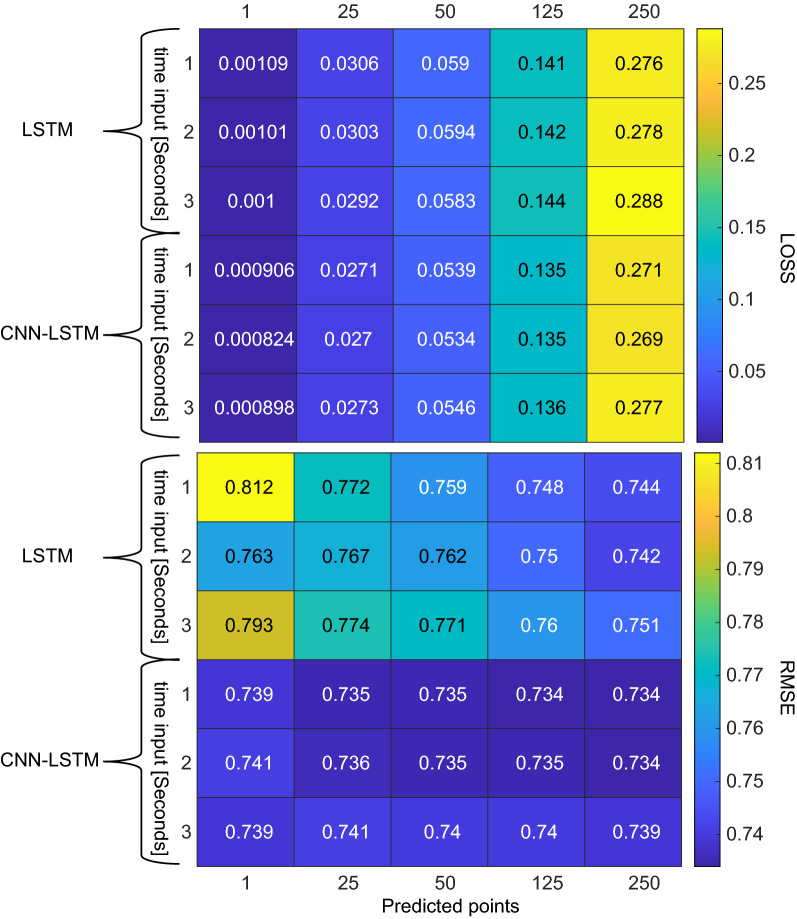
Validation loss (top) and test set RMSE (bottom) of each predicted points (column) vs time input (row) of the LSTM and CNN-LSTM models trained with data set 2

The results of the different models are compiled in Fig. 8, where the validation loss and the RMSE over 1 s of the test set are shown. For the 15 LSTM models, the performance improves with longer output sequences, but are best with 2 s of input. Thus, the best performing model is the 2-s input–1-s output, with a RMSE of 0.7418. In regards to the CNN-LSTM models, performance does not vary significantly across input nor output length; however, the best model is obtained with 1-s input–1-s output which has RMSE of 0.7341. Across all combinations, the CNN-LSTM outperforms the LSTM, as it can extract richer features.

**Table 5:** Performance comparison for forecasting methods

Method	RMSE	Time (s)
LSTM	0.7418	0.0035
CNN-LSTM	0.7341	0.0087
ARMAX	3.1813	0.3645

**Fig. 9:**
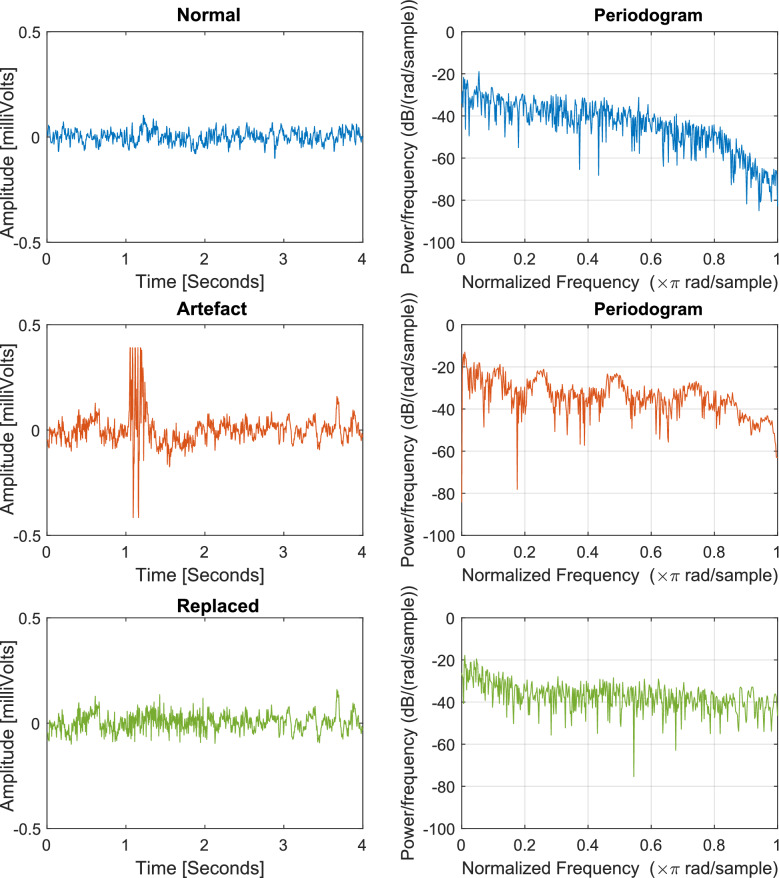
Examples of normal (blue), artefactual (red) and replaced-segments signals (green) alongside their periodogram from data set 2.The method has been able to recreate the normal signal, both in amplitude as in spectral properties

Subsequently, the comparison to the ARMAX model was carried out. The ARMAX was given 1 s of recording to define the model and asked to forecast the subsequent second to calculate the RMSE, achieving a score of 3.1813. The difference in the performance of the ARMAX between the two data sets can be attributed to the fact that the one being evaluated has not been heavily filtered, and retains high-frequency components, making it more difficult to adjust a model. When looking at the overall performance of RMSE and computational time in Table 5, the CNN-LSTM stands out as the best performing method.

With these results, we proceed to extract 4-s (i.e., 1000 data points at 250 Hz) artefactual segments with the condition that the first second had to be artefact-free, for a total of 3826 examples. The forecast produced by the network replaced every 1-s window labelled “artefact” in each segment, which in turn was used as part of the input if the following window also shared the same label. To evaluate the results, examples of the three signals (i.e., normal, artefactual, and replaced-segments signals) with their corresponding periodogram are shown in Fig. 9. Compared to normal segments, artefacts have higher amplitude and frequency, in other words, a non-physiological waveform. We observe this in the periodogram in the repeated round peaks and that the higher frequencies don’t decay as much powerwise. By replacing the segment, the smoothness of the spectrum power decay is returned.

**Fig. 10:**
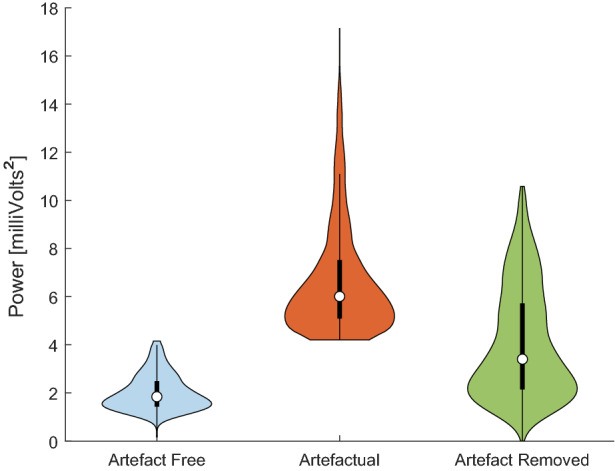
Violin Plot of power in the normal (blue) 1-s segments, artefactual segments before (red) and after (green) processing from data set 2. The method has reduced the power of the artefactual segments to similar values to the artefact-free segments

Finally, the violin plot of the power of the 4-s segments of the three signals is displayed in Fig. 10. Despite the fact that the distribution has lowered significantly to values resembling normal activity, the shortcoming previously mentioned is still present, as cases with surrounding high power are not replaced as they have not exceeded the threshold.

## Conclusion

This paper has presented an artefact replacement algorithm for in-vivo neural recordings in the form of local field potentials. This is particularly useful, where signal segments contaminated with artefacts can not be reconstructed with information from other channels due to the presence of a global artefact or the majority of the channels are affected or the signals are of poor quality (i.e., very low signal-to-noise ratio). This paper introduces a prediction method with the use of a sliding window technique. Two neural networks architectures with recurrent and convolutional layers, along with ARMAX were compared. The best performance was achieved by the CNN-LSTM model. Comparisons were made by observing examples of the classes and the mean power per band across two open-access data sets of LFP signals recorded during different tasks. This revealed that the forecasted data may be used to replace artefact parts successfully in LFP recordings. The model was incorporated into the artefact removal module of the simple and effective SANTIA toolbox is a simple and effective toolbox for researchers who want to automatically detect and remove artefacts.

## Data Availability

The source-code of the toolbox is available at https://github.com/IgnacioFabietti/SANTIAtoolbox.

## Footnotes

1 https://github.com/IgnacioFabietti/SANTIAtoolbox.

2 https://uk.mathworks.com/help/deeplearning/ref/nnet.cnn.layer.layer.html.

3 function extracted from https://github.com/bastibe/Violinplot-Matlab.
